# Regulatory T cells in solid tumor immunotherapy: effect, mechanism and clinical application

**DOI:** 10.1038/s41419-025-07544-w

**Published:** 2025-04-11

**Authors:** Yan Pan, Hanqiong Zhou, Zhenqiang Sun, Yichen Zhu, Zhe Zhang, Jing Han, Yang Liu, Qiming Wang

**Affiliations:** 1 Department of Internal Medicine, The Affiliated Cancer Hospital of Zhengzhou University & Henan Cancer Hospital, Zhengzhou, 450008 China; 2 Institute of Cancer Research, Henan Academy of Innovations in Medical Science, Zhengzhou, 451162 China; 3 Department of Colorectal Surgery, The First Affiliated Hospital of Zhengzhou University, Zhengzhou, 450001 China; 4 Department of Radiation Oncology, The Affiliated Cancer Hospital of Zhengzhou University & Henan Cancer Hospital, Zhengzhou, 450008 China

**Keywords:** Cancer immunotherapy, Immune evasion, Cancer immunotherapy

## Abstract

The tumor-immune response is mobilized to suppress tumorigenesis, while the immune microenvironment and lymph node microenvironment are formed gradually during tumor progression. In fact, tumor surface antigens are not easily recognized by antigen-presenting cells. So it is hard for the immune system to kill the newly formed tumor cells effectively. In a normal immune environment, immune function is always suppressed to maintain the stability of the body, and regulatory T cells play an important role in maintaining immune suppression. However, during tumorigenesis, the suppression of regulatory T cell immune functions is more likely to contribute to tumor cell proliferation and migration leading directly to tumor progression. Therefore, focusing on the role of regulatory T cells in tumor immunity could improve tumor immunotherapy outcomes in the clinic. Regulatory T cells are more mature in hematologic system tumors than in solid tumors. However, there are continuing efforts to apply regulatory T cells for immunotherapy in solid tumors. This review describes the role of regulatory T cells in solid tumor immunotherapy from the perspective of prognosis, immune microenvironment remodeling, and current clinical applications. This summary could help us better understand the mechanisms of regulatory T cells in solid tumor immunotherapy and further expand their clinical application.

## Facts List

- **Tregs as Key Immunosuppressive Agents in Solid Tumors**
- Elevated Treg levels in peripheral blood and tumor tissues correlate with reduced antitumor immune responses and poor immunotherapy efficacy. Tregs produce inhibitory cytokines (e.g., IL-10, TGF-β) and deplete IL-2, suppressing effector T cells (Teff).
- High Foxp3^+^ Treg infiltration paradoxically associates with improved survival in colorectal, esophageal and head/neck cancers but worse prognosis in breast, melanoma and gastric cancers.
- **Treg Subtypes Exhibit Context-Dependents Effects**
- CD4^+^ FoxP3^+^ Tregs dominate immunosuppressive activity in ovarian, lung and colorectal cancers. Depleting CD4^+^ Tregs enhances checkpoint inhibitor efficacy.
- CD8^+^ Tregs are less common but contribute to immune evasion in prostate cancer via indirect suppression mechanisms.
- **TME Interactions Drive Immunosuppression**
- Cancer-associated fibroblasts (CAFs) recruit and retain Tregs via OX40L/PD-L2,
- creating an immunosuppressive microenvironment.
- Hypoxia in solid tumors upregulates HIF-1α and FoxP3, promoting Treg differentiation and TGF-β-mediated suppression.
- **Clinical Interventions Targeting Tregs**
- Anti-CCR4 antibodies selectively deplete Tregs and improve antitumor responses in melanoma and NSCLC.
- Combining low-dose cyclophosphamide with personalized vaccines reduces Tregs and enhances Teff activity in glioblastoma.
- **Contradictory Outcomes in Treg Modulation**
- Pan-Bcl-2 inhibitors (e.g., GX15-070) induce Treg apoptosis but may also affect Teff.
- Dietary interventions (e.g., serine/glycine restriction) reduce Tregs but require optimization to avoid metabolic stress on Teff.

## Open Questions List

- **Mechanistic Heterogeneity Across Tumor Types**
- Why do FoxP3^+^ Tregs improve survival in colorectal cancer but worsen outcomes in breast cancer? What tumor-specific factors dictate this duality?
- **Selective Targeting of Tregs**
- How can therapies specifically deplete or inhibit Tregs without compromising Teff function (e.g., avoiding CD25^+^ Teff depletion)?
- **Treg Plasticity and Metabolic Adaptation**
- What molecular mechanisms allow Tregs to thrive in hypoxic, nutrient-poor TMEs? Can metabolic vulnerabilities (e.g., oxidative phosphorylation dependence) be exploited therapeutically?
- **Combination Strategies to Overcome Resistance**
- Which immunotherapies (e.g., anti-PD-1, vaccines) synergize best with Tregs-targeted agents? How do lymph node stromal cells influence Treg expansion and systemic immunity?
- **Biomarkers for Prognostic and Therapeutic Guidance**
- Are circulation Treg levels or specific TME markers (e.g., CAF-S1) reliable predictors of immunotherapy response? How do Treg subset (CD4^+^ vs. CD8^+^) differ in biomarker utility?

## Background

The tumor immune microenvironment (TIME) contains tumor cells, immune cells around the tumor, and cytokines that regulate tumor immunity. With further investigation of the tumor microenvironment (TME), we found that the TIME is the most important link in immune system clearance of tumor cells and an important factor affecting the prognostic efficacy of tumor immunotherapy [1]. The immune system detects tumor cells by monitoring them. In the early stages of tumor development, the immune system uses DCs to recognize the antigen produced by the tumor and pass the information on to T cells [2]. After receiving these messages, T cells will take the lead in killing tumors. During tumorigenesis, tumor cells affect intrinsic immunity by interacting with the TIME, including DCs, MDSCs, tumor-associated macrophages (TAMs), NK cells, and regulatory T cells (Tregs) [3, 4]. Multiple immune cells in the TIME interact in the microenvironment to construct a suppressive microenvironment. Inhibitory TIME directs tumor cells to evade immune surveillance and promotes invasion and metastasis of surrounding tissues and distant organs. TIME remodeling decreases the effectiveness of some traditional cancer therapies, especially chemotherapies and targeted therapies. However, the development of immunotherapies has improved the efficacy of treatment in refractory, recurrent tumors. When immunotherapies are effective, both overall survival (OS) and progression-free survival (PFS) can be extended [5]. However, further exploration of immunotherapies for tumors and strategies for how to overcome immune resistance in blood systems [6], and the immune environment in solid tumors is needed.

Tregs are a subset of CD4 T cells with immunosuppressive activity and can be classified into natural regulatory T cells (n Tregs) and induced regulatory T cells (i Tregs) depending on their mature site [7]. Unlike T cells, Tregs are a major barrier to tumor immunotherapy, and Tregs are known to play a major role in immune suppression in the TME [8, 9]. Elevated Treg levels were observed in the peripheral blood and tumor tissues of tumor patients [10, 11], a state that resulted in a reduced tumor immune response and reduced efficacy of antitumor immunotherapies. Tregs produce the inhibitory cytokines interleukin 10 (IL-10) and transformed growth factor beta (TGF-β) to regulate the immune response and restrict cytokine availability to effector T cells (Teff) to induce apoptosis [4, 12].

There have been significant advances in the study of how Tregs regulate tumor progression [13]. However, research on how Tregs function in immunotherapies for solid tumors remains limited. In this review, we summarize the prognostic impact of Tregs in solid tumors, mechanisms of action in solid tumor immunotherapy, multiple ways to influence the efficacy of Tregs, and clinical applications of Tregs in solid tumor immunotherapy. Moreover, we discuss the disadvantages of Tregs’ in the current understanding of immunotherapies for solid tumors and suggest possible directions for development in this field.

### Effect of Tregs on solid tumor prognosis

Studies have shown that Tregs play a major role in tumor immunity and promote tumor cell initiation and growth [13]. While Treg number and tumor relationship reports [14] have almost all shown an increased Treg proportion in solid tumors. The emergence of this phenomenon may be due to the presence of hypoxia, low pH, and nutritional deficiencies in the TME. In these particular environments, Tregs are more dependent on oxidative phosphorylation for their unique proliferation and functional advantage than Teff [15, 16]. In a phase II drug study in patients with advanced NSCLC [17], the results showed that dasatinib treatment had an antitumor effect by reducing the number of Tregs and increasing the number of tumor-infiltrating CD8^+^ T cells. Similar findings were also found in established mouse models of breast, colorectal, and melanoma tumors. The findings confirmed that inhibition of Treg cell counts could improve the prognosis of solid tumors. Esther [18] and others compared NSCLC samples with normal lung tissue and found that YES1 expression was the most effective predictor of prognosis in NSCLC patients with SFK. High YES1 protein expression tended to indicate shorter OS, with a significant difference in OS between high YES1 expression and low YES1 expression on the OS curve. High YES1 protein expression was associated with an increased level of tumor-infiltrating Tregs. However, some data [19] show that high FoxP3 Treg infiltration presents a paradoxical relationship with solid tumor prognosis, especially OS. And increased FoxP3 Treg infiltration is positively associated with decreased OS in cervical, kidney, gastric, liver, melanoma, and breast cancers. Conversely, high FoxP3 Treg infiltration is associated with improved OS in esophageal, colorectal, and head and neck tumors [19]. Specifically, FoxP3 Tregs showed prognostic effects in breast and ovarian cancer, where FoxP3 Tregs were associated with estrogen receptor (ER) status. High FoxP3 Tregs infiltration in ER^-^ breast cancer patients were associated with better outcomes, while it was associated with poor prognosis in ER^+^ patients with the same status. In ovarian cancer, FoxP3 Tregs were strongly associated with disease stage, and increased infiltration of FoxP3 Tregs in advanced or highly differentiated ovarian tumors suggested improved survival [19]. These findings and conclusions suggest that Tregs may play a role in the prognosis of solid tumors in different organs, and that the mechanism of action of specific site-specific solid tumors needs to be further explored.

### Effect of different types of Tregs on solid tumor treatment

Tregs are classified into different types depending on the antigen markers on the cell surface. In the upcoming discussion, we will analyze the efficacy of different types of Tregs (based on CD4 and CD8 expression) in solid tumor treatment.

The first subtype of Tregs is CD4^+^ Tregs. CD4^+^ Tregs play an anti-inflammatory role in the maintenance of autoimmunity. In an article investigating the phenotype of tumor-infiltrating lymphocytes (TILs) in ovarian cancer, researchers showed [20] that CD4^+^ TILs in high-grade serous ovarian cancer (HGSOC) consists mostly of Tregs. In HGSOC, triple-positive CD4^+^ Tregs had higher immunosuppressive activity [21]. From the perspective of HGSOC samples, triple-positive HGSOC is mostly derived from malignant samples. The high immunosuppressive activity of CD4^+^ Tregs has also been described in both lung and CRC [22]. During tumorigenesis, FoxP3^+^ Tregs inhibited antitumor immunity, promoted tumor escape and progression, and decreased immunotherapeutic efficacy. In solid tumors such as lung, melanoma, and esophageal cancer, high infiltration of FoxP3^+^ Tregs in tumor tissues was detected, and an increased immune response was observed after selective depletion of FoxP3^+^ Tregs using different immunotherapies [23–25]. These results suggest that specific depletion of CD4^+^ CD25^+^ FoxP3 Tregs may improve the efficacy of solid tumor immunotherapies, such as anti-human CCR4 monoclonal antibody exerting antibody-dependent cytotoxic activity (ADCC). Moreover, the depletion of CD4^+^ CD25^+^ FoxP3 Tregs improves the efficacy cancer immunotherapies, such as checkpoint inhibitors, enhances the immune response and prolongs the survival of tumor patients [24].

The second subtype of Tregs is CD8^+^ Tregs. Most CD8^+^ Tregs are generated by antigen stimulation. CD8^+^ CD28^−^-Tregs can stimulate peripheral induction through MHCI-like peptides. The mechanism consists of the following two aspects: a. prioritizing the expression of FoxP3α subtypes leading to immune suppression of the indirect cellular mechanisms of contact [26–28]; b. directly targeting antigen presenting cells (APCs) to upregulate the inhibitor receptor ILT3/4 to mediate antitumor immune suppression [29, 30]. In prostate cancer immunosuppression studies, CD8^+^ CD25^+^ Tregs were detected at tumorigenic sites [31]. CD8^+^ CD25^+^ Treg cell surface markers were similar to those of CD4^+^ Tregs, mainly through the cell indirect exposure mechanism or IL-4 inhibitory T-cell proliferation and efficacy [32, 33]. Therefore, CD8^+^CD25^+^-Tregs are phenotypically and functionally similar to CD4^+^CD25^+^ Tregs, but different CD8^+^ Tregs are more susceptible to the TME and are more immune to treatment [31, 32].

**Fig. 1:**
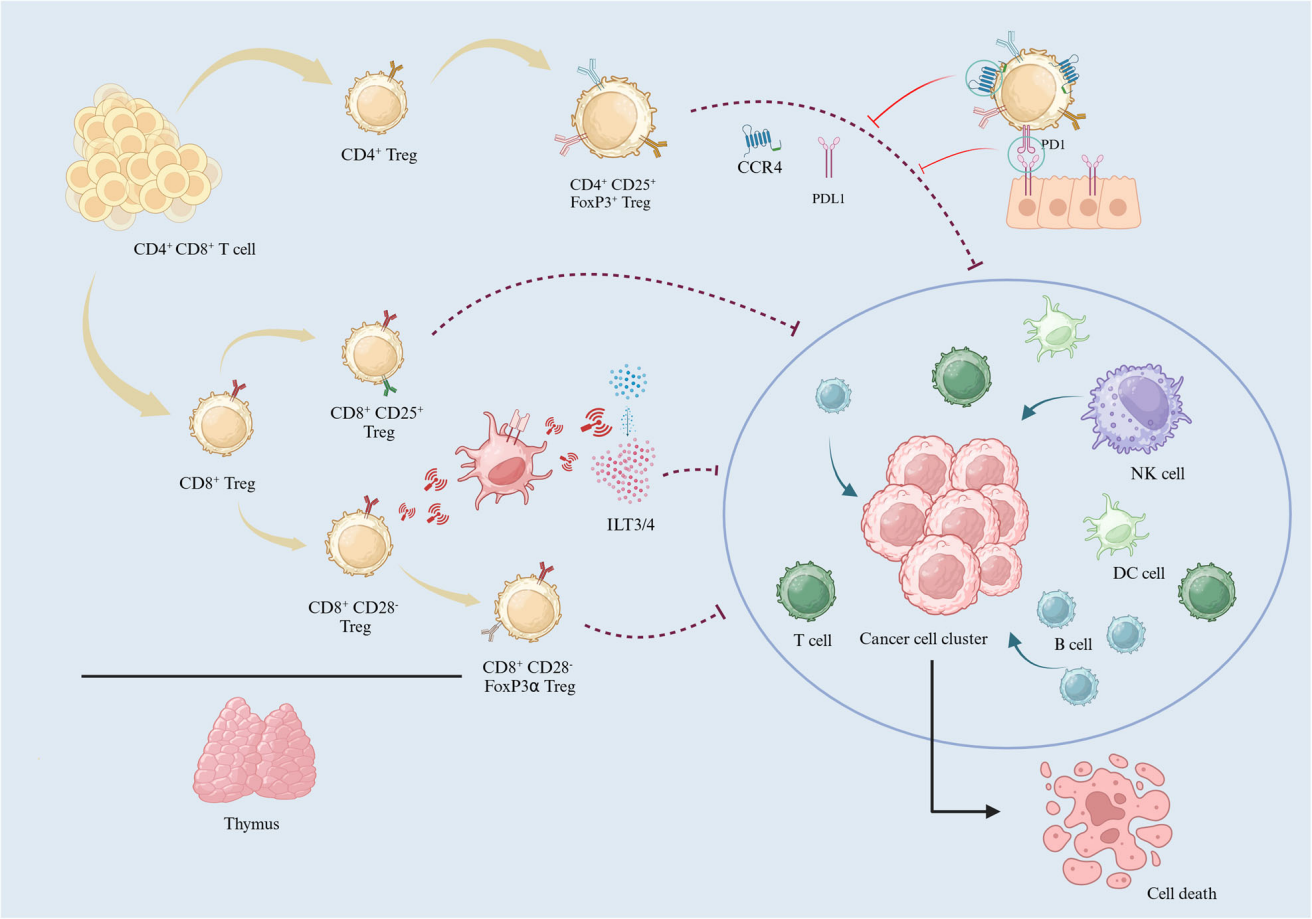
Effect of different types of Tregs on solid tumor treatment. Tregs with different antigens display different ways to influence immune cells kill cancer cells.

Overall, Tregs are mostly CD4^+^ cells and mostly exhibit antitumor immune suppression effects. Thus, eliminating Tregs and enhancing the antitumor immunity of the body or the efficacy of immunotherapeutic agents is necessary to improve the response to immunotherapy. CD4^+^ Tregs are highly infiltrated and detectable in the peripheral blood in breast cancer, cervical cancer, prostate cancer, hepatocellular carcinoma, melanoma and many other types of tumors [25, 34, 35], and they can be detected in human peripheral blood. However, CD8^+^ Tregs are often only cultured and induced in the TME, and their mechanism of action varies for tumors at different sites. For example, when subcutaneous tumors exhibit depletion, while lung tumors exhibit high levels, resulting in increased tumor volume [36]. These results suggest that to some extent, the specific role of Tregs varies for different types of solid tumors. Overall, Tregs induce Treg cell production in the TME, leading to the formation of a locally immunosuppressive microenvironment [37]. Understanding how tumor sites induce Treg production and depletion of established Tregs is therefore critical to enhancing immunotherapeutic efficacy (Fig. 1).

### Interaction of Tregs with other immune cells influences immunotherapeutic efficacy

In the TIME, Tregs, whose primary role is to suppress immunity, affect other types of immune cells through a variety of mechanisms. Here, we examined two of the most iconic immune cell types, CD8^+^ T cells and cancer-associated fibroblasts (CAFs).

First, we introduce the interactions between Tregs and CD8^+^ T cells. As the tumor progresses, Tregs account for an increasing proportion of the TME. Meanwhile, the levels of T cells in general, and CD8^+^ T cells in particular, increase first and then decrease. Therefore, a decrease in the ratio of CD8^+^ T cells to Tregs is also considered an important indicator of a poor prognosis in solid tumors. Single-cell sequencing revealed T-cell immune receptor (TCR) library alterations in tumor-associated CD8^+^ T cells, reduced clonal diversity and selective clones in advanced-stage tumors vs. early-stage tumors in both murine melanoma and lung adenocarcinoma models. There was a modest restoration of clonal diversity in CD8^+^ T cells following selective depletion of Tregs, suggesting that Tregs contribute to Treg library remodeling in the TME as tumors develop. IL-2 is deficient in the TME, especially at an advanced stage. The research team found that supplementing with IL-2 would promote retention and functional expression of Teff in the TME [38], reducing the intra-tumoral Treg ratio [39], thus increasing the CD8^+^ T to Treg ratio in favor of a lower probability of poor prognosis. Some reports [40] suggest that the combination of immunotherapeutic agents such as ipimumab with Ip2 could effectively activate tumor-infiltrating lymphocytes (TILs) to induce systemic immunity. However, the relative contribution of this combination in treatment models is not explained.

Aside from CD8^+^ T cells, Tregs also interact with CAFs. Fibroblasts are an integral part of the TME: normal fibroblasts inhibit tumorigenesis [41], and CAFs enhance the proliferation and invasiveness of cancer cells and promote angiogenesis [42, 43]. Fibroblasts have also been shown to inhibit antitumor immunity and aid in the acquisition of resistance in tumor tissue [44–47]. In human breast cancer fibroblasts, enrichment of CAF-S1 subsets in breast tumors is associated with accumulation of FoxP3^+^ Tregs. Among tumor-secreted cytokines, CAF-S1 is significantly associated with IL-17F, IL-1β, IL-10, and IL-6, which inhibit T-cell activation [48, 49]; the infiltration levels of FoxP3^+^ Tregs tend to predict a poor prognosis in tumors. The same results were found in pancreatic cancer [50]. Further study of CAF-S1 in interaction with CD4^+^ CD25^+^ Tregs revealed that CAF-S1 may retain Tregs through key factors such as OX40L, PD-L2 and JAM2, suggesting that the CAF-S1 subpopulation has a certain ability to attract and retain Tregs. On the one hand, CAF-S1 increases the number of Tregs in the TME. On the other hand, CAF-S1 inhibits the proliferation of Teff. These abilities all contribute to tumor initiation and progression by supporting the development of a TME that suppresses the immune response [48].

### Tregs influence the mechanisms of immunotherapy in solid tumors

**Fig. 2:**
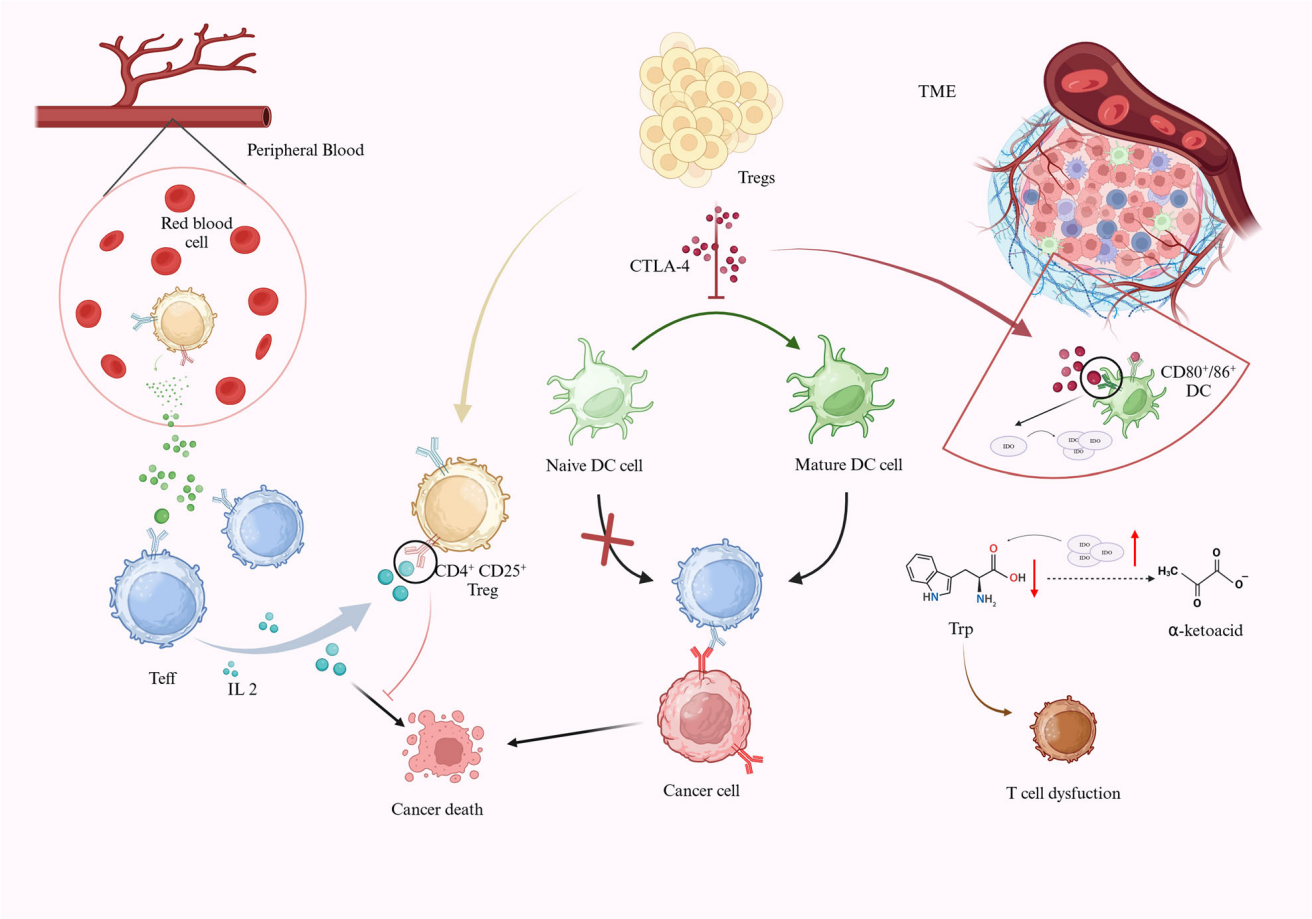
Tregs influence mechanisms of immunotherapy in solid tumors.

The advent of immunotherapies has greatly improved the efficacy of tumor therapy. However, they have only interfered with tumor progression in a small subset of patients, suggesting that there may be unknown mechanisms of immune suppression in the immunosuppressive TME [51–53]. Tregs play an important role in inhibiting the immune response and facilitating tumor immune escape in solid tumors, and the mechanisms that influence immunotherapy are classified as follows (Fig. 2):

1. Mechanisms mediated by cytokines: Some researchers have previously shown [54] that Tregs are derived primarily from a subset of CD4^+^ T cells and mostly express CD25. CD4^+^CD25^+^ Tregs competitively suppress the immune response to tumor cells by binding the IL-2 high affinity receptor to IL-2, and IL-2 is one of the cytokines of the primary stimulatory production of Teff [54, 55]. Moreover, CD25^+^ Tregs in peripheral blood express perforin and granzyme B, both of which kill effector T cells and thus mediate tumor immune suppression [56, 57].
2. Mechanisms influencing DC antigen presentation: Tregs express cytotoxic T-cell antigen 4 (CTLA-4), affecting tumor surface antigen maturation, and immature anti-CTLA-induced T cells fail to recognize tumor surface antigens, leading to tumor cell immune escape. In melanoma models, to some extent, increased immune activity after anti-CTLA-4 treatment improved tumor prognosis [58–61].
3. Mechanisms mediated by metabolites in the TME: Tregs expressing CTLA-4 interact with CD80/86 on the surface of antigen presenting cells to promote the secretion of indoleamine 2,3-dioxygenase (IDO). IDO is an enzyme essential for tryptophan metabolism. For tryptophan, its overexpression in the TME leads to T-cell dysfunction, which suppresses the T-cell immune response against tumors [62, 63].

### Tregs influence the efficacy of solid tumor immunotherapies by reshaping the TME

The immunosuppressive TME is an important marker of tumor emergence and the basis of resistance and immune escape after immunotherapy [64]. Multiple studies have shown that Tregs play a key role in tumorigenesis and progression. Treg recruitment in tumor tissue mediates the formation of a tolerant TME, redirects the body from a stable immune state to an immunosuppressive state, and inhibits the body’s immune activity against tumors [65, 66]. The coregulation of these mechanisms contributes in part to immune escape [67, 68]. One study [39] showed that Tregs accumulated in the TME as the tumor progressed in murine melanoma and lung adenocarcinoma models. Its increase was associated with higher expression of TCRs in B and T lymphocyte attenuators (BTLA) and PD-1 on the surface of tumor-infiltrating CD8^+^ T cells. Especially in advanced disease, increased infiltration of Tregs is associated with increased expression of immune receptors and decreased immune function.

Although Tregs are the primary inhibitory cells in the immunosuppressive TME, there are many other regulatory cells that together contribute to the formation of the immunosuppressive TME. In some tumor tissues, such as breast cancer [69], pancreatic cancer [70], and glioblastoma [71], tumor-associated macrophages (TAMs) are also components of the immunosuppressive TME [72, 73]. Tregs with Nrp1 deficiency are known to lose their ability to inhibit the tumor immune microenvironment, which means that Nrp1 deficiency enhances antitumor activity [74]. In mouse melanoma and colon adenocarcinoma models for studying Treg mechanisms of interfering with tumor-driven immune suppression reprogramming, researchers found [75] that both models contain downstream SREBP1 pathways. The same downstream pathway suggests that Tregs may have a conservative downstream regulatory mechanism for different solid tumor types, leading to a broad impact on immune transcription leading to antitumor immunity. In subsequent studies of Treg regulatory transcription mechanisms, researchers found that Treg dysfunction may lead to alterations in bone marrow cells. For example, macrophages metabolic pathways in the immunosuppressive TME suggest that Tregs modulate TAMs by affecting lipid metabolism through the SREBP1 pathway. After using wild-type Tregs and Nrp1^L/L^ Tregs to induce TAMs in the TME, researchers found that Nrp1- induced phenotype identification in M2-like mice with a reduced immune response to the tumor-like TME [75–77].

### Tregs influence the response to solid tumor immunotherapies through the lymph node microenvironment

Lymph nodes provide a relatively restricted environment for initiating an adaptive immune response, continuous immune surveillance, and immune tolerance in the body at the onset of disease.

Immune cells, especially T cells, in peripheral tissues rapidly travel through the lymphatic system to the lymph nodes surrounding the lesion [78]. As soon APCs are activated, proliferate, and differentiate, the antigen signal is recognized in the lymph nodes [78, 79]. In a stable state, immune function in lymph nodes is usually suppressed when stromal cells in lymph nodes are able to induce the differentiation of juvenile CD4 T cells into Tregs through MHC-II expression and autoantigen presentation [79, 80]. After tumorigenesis, due to reduced fluid flow [81], circulating tumor cells (CTCs) have increased lymphatic system retention, which may activate YES-related protein 1 (YAP1) and transcriptional coactivators with PDZ-binding motif (TAZ) signaling pathways in CTCs. Therefore, YES1 protein expression is positively associated with the tumor-infiltrating Treg population [18, 81].

In mouse melanoma models, genes that regulate the fatty acid oxidation pathway were found to increase fatty acid content. When the lymph node microenvironment is rich in fatty acids, metastatic tumor cells activate YAP-dependent metabolic pathways. This process leads to increased adaptive and transmissible capacity of metastatic tumor cells and reduced body survival. The positive association between tumor-infiltrating Tregs and YAP expression is also consistent with increased tumor invasion and reduced survival of Tregs in response to tumors [18, 82].

Tregs, on the other hand, help tumor cells evade immune recognition by inhibiting lymph node microenvironment functions, such as antigen delivery. Tumor cells generally do not express MHC on the surface and cytotoxic T cells are difficult to recognize. Therefore, peripheral lymph nodes often fail to stimulate T-cell activation and induce Treg production, which first suppresses the establishment of an antitumor immune response and then leads to tumor antigen resistance [48, 83, 84]. The immune function state remains immunosuppressive in lymph nodes due to the inability of the body to recognize tumor cell surface antigens. Lymph stromal cells such as lymph node endothelial cells (LECs) help tumor cells complete immune escape by promoting the expansion of CD4^+^ CD25^-^ Tregs with PD-L1 or NO and inhibiting the proliferation of other T cells [80, 85, 86].

**Fig. 3:**
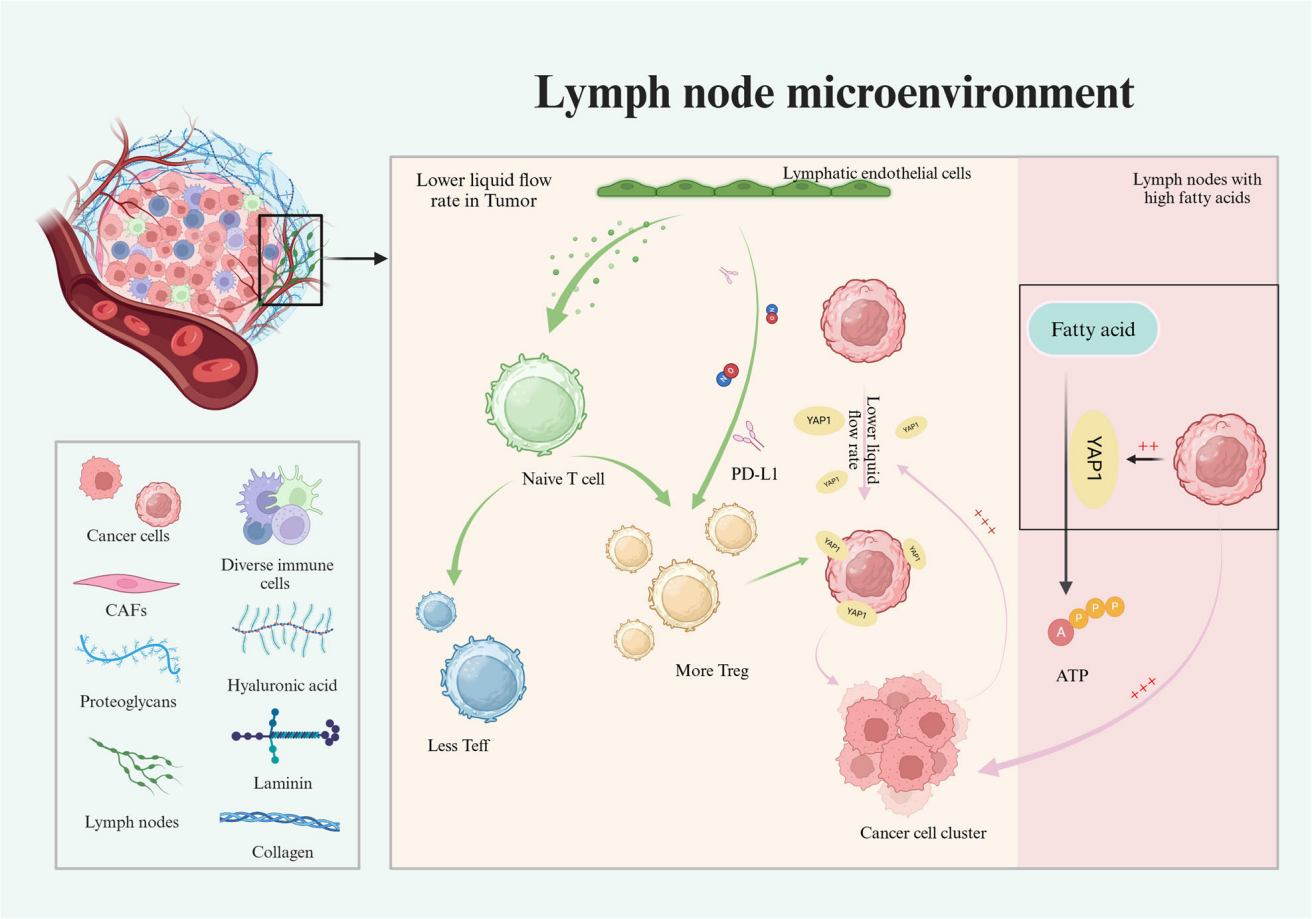
Tregs influence the response to solid tumor immunotherapies through the lymph node microenvironment. Lower liquid flow in lymph node microenvironment makes YAP1 combine with cancer cell so that the liquid rate gets more lower. YAP1 conduct fatty acid metabolism, cancer cells join this course and make itself multiplication.

In general, the lymph node microenvironment remains immunosuppressive at the time of solid tumor onset due to the difficulty of recognizing the surface antigen of the tumor cells. The combination of tumor cells acting on the lymph node microenvironment promotes Treg proliferation, which drives immune escape and suppresses the body’s immune response, leading to the formation of a lymph node microenvironment conducive to tumor growth, invasion and migration (Fig. 3).

### Different agents modulate Tregs to affect solid tumor immunotherapy efficacy

In the course of immunotherapy for solid tumors, the therapeutic effect decreases gradually with the increase in the level of Treg infiltration. How to reduce the number or inhibit the activity of Tregs is one of the difficulties to overcome in immunotherapy for solid tumors.

The first influencing factor we will discuss is pan-Bcl-2 inhibitors. The Bcl-2 family of genes inhibits apoptosis by modulating endogenous pathways that ultimately lead to tumorigenesis, so using Bcl-2 inhibitors to reduce Bcl-2 expression is one option for antitumor therapies. In terms of clinical advantages, Bcl-2 inhibitors have been shown to be uniquely safe and well established in the treatment of hematologic malignancies such as chronic lymphocytic leukemia (CLL), diffuse large B-cell lymphoma (DLBCL), mantle cell lymphoma (MCL), and follicular lymphoma (FL) [87]. However, their efficacy in solid tumors is limited. Research [88] found that the pan-Bcl-2 inhibitor GX15-070 (Obatoclax) induced apoptosis in Tregs, significantly decreased FOXP3 and CTLA-4 expression in Tregs and suppressed the inhibition of both in response to drug dosing. A study [88] showed that Tregs are more sensitive to GX-15 than CD4-Teff in peripheral blood monocytes from ovarian cancer patients, so GX-15 significantly inhibits Treg function with little or no impact on Teff function. GX-15 can enhance the antitumor activity of our body and vaccine-mediated antitumor efficacy [89]. GX-15 is an ideal combination therapy, especially when immunotherapy is the primary treatment.

The second most important factor is hypoxia. Intra-tumoral hypoxia is found in almost all solid tumors. Poor clinicopathologic type and tumor-associated malignant events often occur, and hypoxia in the TME may lead to increased tumor immune suppression, poor response to antitumor drugs, accelerated malignant progression, and local infiltration [90, 91]. In a study of HIF-1α and the Treg surface marker Foxp3 in gastric cancer [92], expression of HIF-1α and Foxp3 was found to be positively correlated with tumor stage and degree of peripheral lymph node metastasis. Both HIF-1α and Foxp3 levels were higher in advanced metastatic gastric cancer tissues than in nonmetastatic gastric cancer tissues. OS was significantly higher in the Foxp3 low expression group than in the Foxp3 high expression group. The HIF-1α low score group was significantly higher than the HIF-1α high score group. The researchers also found an increase in mRNA expression of the immunosuppressive cytokine TGF-β1 under hypoxic conditions and increased expression of HIF-1α in gastric cancer cell lines [92–94]. T cells showed increased Foxp1 expression in recombinant human TGF-β3 and hypoxic media compared to the control group, and Foxp3 expression was significantly higher in hypoxic media than in the normal oxygen content groups. These results demonstrate that hypoxia in gastric cancer cell lines enhances the ability to induce regulatory T cells via TGF-β1, leading to enhanced tumor immunosuppression and accelerated progression.

The last significant factor to be discussed is arsenic trioxide (As_2_O_3_). As_2_O_3_ showed strong antitumor efficacy, especially in patients with acute promyelocytic leukemia (APL) [95]. The same group also found that As_2_O_3_ enhances the immune response to breast cancer cells, myeloma cells and colorectal cancer cells [96–98], reflecting the strong activity of arsenic trioxide in tumor cells involved in immune suppression in the body. Among these tumors, colorectal cancer cells were regulated primarily by selective oxidation of low doses of As_2_O_3_ (0.5 μM or 1 μM) and nitrite burst-depletion of Tregs [98]; the immune response of the body increased and mice with higher immune activity experienced more significant tumor growth delays after treatment with a single dose of As_2_O_3_. This trait may be related to the redox state of the cells, which are more likely to meet cytotoxic thresholds after exposure to As_2_O_3_ due to higher levels of peroxynitrite ion (ONOO-) bases in Tregs [98]. Overall, As2O3 slows the growth of solid tumors by modulating the depletion of Tregs, improves the immune response of the body to tumor cells, and provides new ideas and opportunities for immunotherapy in solid tumors.

### Tregs affect the response of solid tumors to vaccine immunotherapy

Tregs exert antitumor immunity [13], and Treg depletion targeting the IL-4 receptor (CD25) has been shown in several preclinical models to restore antitumor activity in the tumor immune microenvironment [99–102]. However, since CD25 is not a Treg surface-specific marker receptor, CD25^+^ Teff are also damaged when anti-CD25, resulting in failure to drive antitumor immunity [103–105]. The development of second-generation sequencing technology has enabled the identification of tumor surface-specific antigens, which could facilitate the development of specific anticancer vaccines [106–108]. The combination of an anticancer vaccine that induces selective Treg depletion could improve the efficacy of cancer immunotherapy. BALB/c FoxP3 Dtr transgenic mice were used to study selective Treg depletion in combination with an anticancer vaccine. The results showed that DTX depleted Tregs briefly and this depletion occurred again after CD8 T-cell populations recovered for multiple drug use. In kidney and CRC models, DTX-mediated Treg depletion resulted in complete tumor regression and no recurrence, while DTX-mediated selective Treg depletion in combination with peptide vaccine increased tumor growth even in models with minimal tumor growth and delayed survival [109]. A clinical trial shows personalized DC vaccine combined with the Treg exhaustion can improve the treatment effectiveness [110]. The staffs designed a tumor-associated antigens (TAAs)-based personalized DC vaccine to enhance the CD4^+^ and CD8^+^ T cells in Glioblastoma multiforme and NSCLC. However, this will also enhance the number of Tregs. So the staffs combined their vaccine with low-dose cyclophosphamide to reduce Tregs and improve the effectiveness of the TAA-based the personalized DC vaccine [110]. These results suggest that selective Treg inhibition plays an important role in enhancing the activity of anticancer vaccines and enhancing the efficacy of immunotherapy in solid tumors.

### Treg functions in different solid tumors

**Fig. 4:**
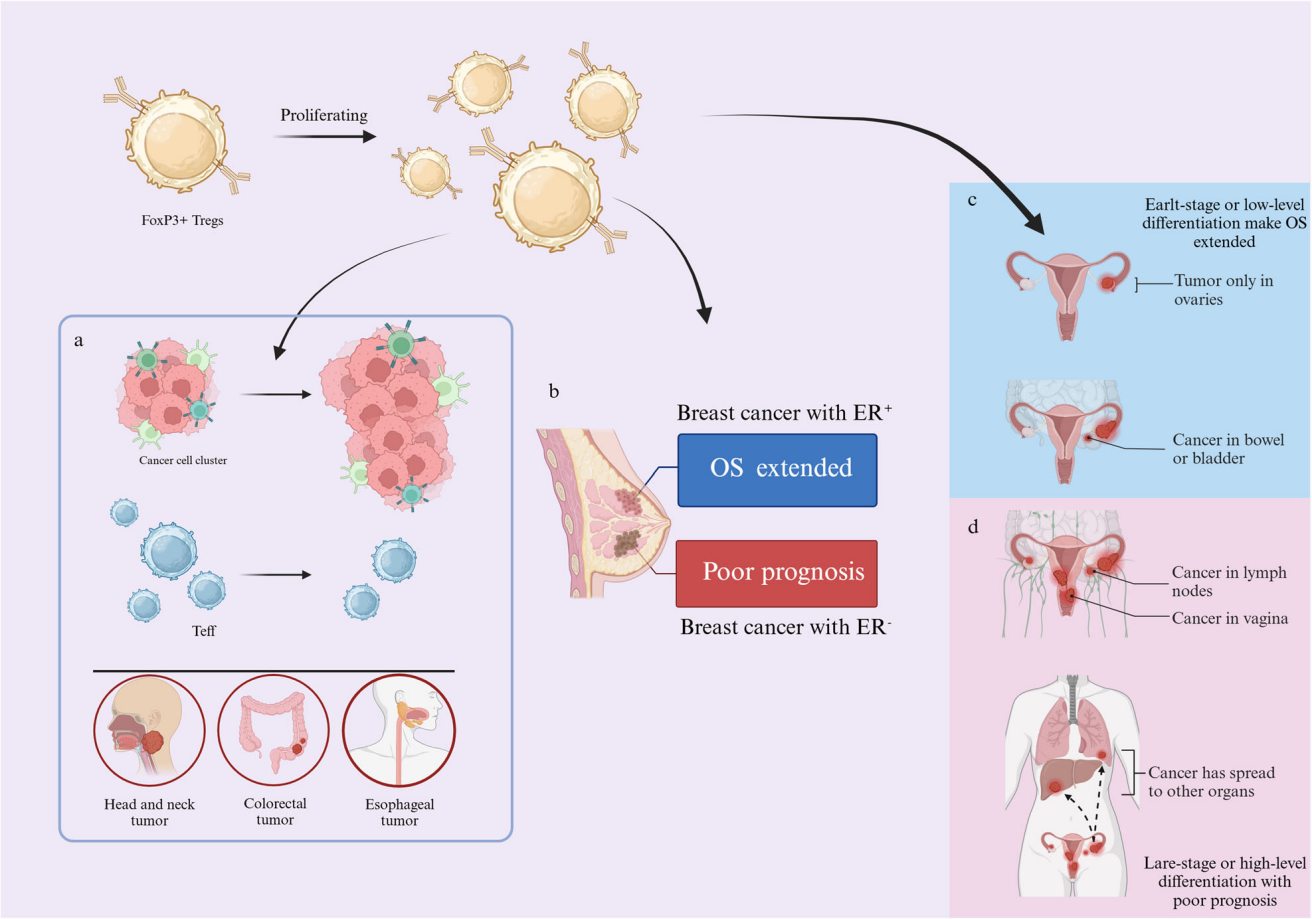
Tregs functions in different solid tumors. **a** FoxP3^+^ Tregs make some kinds of tumor have poor prognosis. **b**–**d** In different types or different stages, Tregs display different roles.

Tregs play a major role in inhibiting the immune response of the body during tumorigenesis and remain negatively regulated for OS in cancer patients. However, Tregs have different prognostic effects depending on tumor type or stage [19]. In tissues with high levels of FoxP3^+^ Tregs, there was a general trend towards higher infiltration and lower OS. In ovarian and pancreatic cancers, the association was not strong, however, in colorectal, head and neck, and esophageal cancers, higher infiltration showed an anomalous increase in OS [19, 111, 112]. Treg infiltration and prognosis were also different for the same cancer species, different subtypes or different stages. For example, in breast tumors, different states of ER caused Tregs to have different prognostic effects, mainly when FoxP3^+^ Tregs were highly infiltrated, while ER^-^ breast cancer had a good prognosis and ER^-^ breast cancer had a poor prognosis [19, 113, 114]. For tumors at different stages, in the case of ovarian cancer, survival was improved with high Tregs infiltration in higher or later stages, while the extent of Treg infiltration at other stages remained inversely correlated with tumor prognosis [19, 115]. In summary, the differential prognostic performance of FoxP3^+^ Tregs in solid tumors may be due to differences in the biological characteristics of different tumor types or within the same tumor type(Fig. 4) [19]. Thus, specific studies of solid tumors at difference stages may facilitate the application of Tregs in improving immunotherapy outcomes.

### Clinical application of Tregs in solid tumor immunotherapy

As research into the TIME has advanced, researchers have gained a better understanding of Tregs cell’s role in cancer immunotherapy. In recent years, researchers in various countries have conducted a series of clinical trials about Tregs cell’s usage in solid tumor, some of them have reached staggered results (Table 1).

**Table 1:** Application of Tregs in clinical solid cancer immunity (Data source: ClinicalTrials.gov: https://beta.clinicaltrials.gov/ provided by the U.S. National Library of Medicine).

Cancer type	Application phase	Purpose of application	Clinical Trial No
Benign Ovarian Tumor/Borderline Ovarian Tumors/Ovarian cancer	Completed	Estimating the percentage and of Treg, Th17 and NKT in peripheral blood and the tissue of the epithelial ovarian tumor and relationship with blood serum level of HE4, CA125, as well as algorithm ROMA.	NCT03779399
Colorectal cancer	Completed	Eliminating cancer tumor using an autologous cell therapy aiming at mounting an efficient immune anti-tumor response by selectively depleting regulatory T-cell during a controlled amount of time.	NCT00986518
Glioblastoma	Phase II	DC migration study to evaluate Treg depletion in GBM patients with and without varlilumab (DERIVe).	NCT03688178
Metastatic Renal Cell Carcinoma	Unknown	Identifying Nivolumab predictive biomarkers in metastatic renal cancer patients through functional evaluation of peripheral Tregs and NKs; the efficacy of new CXCR4 antagonists will be ex vivo evaluated in modulating Tregs and NKs function.	NCT03891485
Hepatocellular Carcinoma	Unknown	Determining the enumeration and function changes of regulatory T cells in peripheral blood of hepatocellular carcinoma patients before and 1 week, 4 weeks after ablation therapy.	NCT01668381
Head and Neck Cancer Squamous Cell Carcinoma/Non-small Cell Lung Cancer/Gastric Cancer	Terminated	Evaluated ADCT-301 in patients with selected advanced solid tumors.	NCT03621982
Cholangiocarcinoma	Unknown	Phase I study of the adoptive immunotherapy for solid tumors using modified autologous cytokine-induced killer cells.	NCT01868490
Glioblastoma/Glioma	Completed	Determined the influence of bevacizumab treatment on circulating immune cells in high grade glioma patients and to search for a link between the variation of these cells and the response to treatment.	NCT01836536
Platinum-resistant Ovarian Cancer/Hepatocellular Carcinoma/Colorectal Cancer/Renal Cell Carcinoma/HER2 Negative Breast Cancer	Recruiting	Using GI-102 as a single agent and in combination with conventional anti-cancer drugs, pembrolizumab or trastuzumab deruxtecan(T-DXd) over a range of advanced and/or metastatic solid tumors. GI-102 can block CTLA-4 expressed on the Treg cells.	NCT05824975
Epithelial Ovarian Cancer	Recruiting	This study will use dose escalation and dose expansion to investigate the safety and efficacy of a combined regimen of pembrolizumab with regulatory T cell depletion and E7777(denileukin diftitox) in patients diagnosed with recurrent or metastatic solid tumors in the second line setting.	NCT05200559

*Treg* regulatory T cell, *NKT* natural killer T cell, *ROMA* Risk of Ovarian Malignancy Algorithm, *DC* Dendritic cell, *GBM* Glioblastoma, *CXCR4* Chemokine (C-X-C motif) receptor 4, *CTLA-4* cytotoxic T lymphocyte-associated antigen-4.

With the development of immunotherapies, improving immunotherapies in solid tumors is an important issue. There are currently five main types of immune otherapies for tumors [116, 117]: a. Molecular targeted therapy; b. immune checkpoint inhibitors, including PD-1/PD-L1 and CTLA-4 inhibitors; c. secondary immune cell therapy, mainly reprogramming and re-transfusion of CAR-T, TIL, NK, CIK/DC-CIK cells. d. cytokine therapy; e. cancer vaccines.

However, immunotherapies are not effective in most patients with metastatic tumors, and there are significant drug side effects that cannot be ignored [118], so a number of combination therapies have emerged to improve tumor immunotherapies. In the clinical treatment of metastatic melanoma and bladder tumors, there is an adenovirus-based CD40 ligand (AdCD40L) gene therapy in which CD40L interacts with CD40 to promote an adjuvant T-cell 1 (Th1) immune response, leading to T-cell activation and migration to the TME [4, 119, 120]. Local applications of AdCD40L inhibit Treg production while promoting Teff to improve the long-term immune response [121]. In clinical trials of combination therapy in ovarian tumors, the Pan-Bcl-2 inhibitor GX15-070 (Obatola) was found to significantly downregulate FoxP3, a Treg cell-specific marker. Tregs were more sensitive to the drug than CD4^+^ T cells, suggesting that GX15 specifically inhibits the function of Tregs and that the combination of GX15 treatment with antitumor vaccine results in better antitumor efficacy [88]. Similarly, inhibition of Treg function to reduce their number was associated with improved OS in patients with metastatic kidney cancer treated with sunitinib [122]. In a recent clinical trial, the researchers use an immunotherapy consisting in autologous lymphocytes infusion depleted from Tregs: patients will be treated with a chemotherapy in first state (5 days), the drugs include cyclophosphamide (day 1 & 2) and fludarabine (day 1 to 5). After these treatments, patients will be infused autologous lymphocytes which depleted the Tregs. The researchers will monitor the tumors size by computed tomography (CT)-scan (ClinicalTrials.gov ID NCT00986518). In another clinical trial, the researchers, Tregs’ function are used to determine a new drug’s efficiency. Nivolumab, a human IgG4 anti-PD1 monoclonal antibody, was permitted to treat the patients who have advanced clear cell renal cell carcinoma (ccRCC). CXCR4 ligand can control the immune cells get into tumors. Tregs suppress nearly all immune cells include NK cells and 70% RCC patients have von Hippel-Lindau (VHL) gene mutations [123], this gene can promote NK-cell function, and its function is a crucial element in nivolumab sensitivity. The researchers monitor Tregs and NK cells’ function from RCC patients’ peripheral blood or neoplastic tissue who undergoing nivolumab treatment, then detect ex vivo effect of CXCR4 antagonists and other Tregs targets antagonists or agonists as anti-PD1 resistance mechanisms (ClinicalTrials.gov ID NCT03891485).

How to reduce the production of the Tregs is a center concern in clinical immunotherapy. There are an increasing number of clinical trials have shown that suppressing the production of Tregs will attain improved treatment effects in solid tumors. A clinical trial in phase 1a of Treg depletion by using mogamulizumab (KW-0761, anti-CCR4 mAb) shows that this drug can significantly decrease the enhanced Treg in peripheral blood [23]. Nevertheless, mogamulizumab is limited efficacy in clinical application because the consumption of CD8^+^ T cell will also be discovered [124]. Recently, another clinical trial demonstrates that adjust the dietary structure can augment antitumor immunity. The results [125] show that the serine/glycine-free diet (-SG diet) can enhance the infiltration of T cells and B cells into tumors with minor impact on T cells’ function in CRC patients. In this study, the staffs analyzed the TIME and found that naïve T cells and Teff are rising sharply while the Tregs decreasing significantly in -SG diet group [125]. Meanwhile, the PD-1 is highly expressed in the increased T cells’ proportion. The combination of PD-1 inhibitors and -SG diet markedly reduce the growth of tumor and the volume of the tumors [125]. Obviously, -SG diet can clearly decrease the number of Tregs and enhance the function of other kinds T cells. There are another clinical trial [126] shows that enhancing the number of Teff and keeping or reducing the Tregs will help improve the function of ICIs. So in other solid tumors, we can use the similar way to aid in improving the outcomes of immunotherapy in clinical practice.

Therefore, in current clinical trials, antitumor drugs that inhibit Treg function tend to achieve better clinical outcomes than other drug types, especially when used in combination with other immunotherapies, such as antitumor vaccines, resulting in significant improvements in OS and long-term outcomes (Table 1).

## Conclusion

In brief, we conclude that Tregs affect the efficacy of immunotherapy in solid tumors through a variety of mechanisms and modes. Tregs continually exert immunosuppressive activity in tumor immunotherapy. Immunosuppression of Tregs contributes to tumor immune escape, aids tumor microenvironment and lymphatic microenvironment shaping in favor of tumor cells and is a major challenge in tumor immunotherapy. Almost all clinical trial displayed that the reduction of Tregs will be so beneficial to other kinds T cells that T cells can perform better cellular immune function. The reason for poor efficacy is that solid tumors are localized and heterogeneous in their tumor cell development across tumor types. Immunotherapies, especially immune-regulatory therapies, are far less effective for solid tumors than for hematologic tumors. It is necessary to further explore mechanism of action of Tregs in nonhematologic tumors and to explore how the tumor immune microenvironment is constructed to enhance the efficacy of single immunotherapy or combination therapy in solid tumors. Antitumor vaccines and anti-Treg combinations have been shown to improve the efficacy of immunotherapy in solid tumors, but the mechanisms by which Tregs influence immunotherapy across tumors are not yet completely understood. Moreover, the efficacy of the combination vaccine is still limited, so increasing the efficacy of the vaccine to inhibit Treg cooperation could be a new way to enhance the efficacy of immunotherapy in solid tumors in the future. In this review, the methods we mentioned, such as modifying the dietary composition and personalized vaccines, can be combined in future experiments. Or the researchers can consider the combination of enhancing the Teff’s function through cytokines like IL2 and decreasing the number of Tregs to amplify the effects of immunotherapy. Cancer outcomes vary by tumor type and location, and this is an important issue to be addressed in future studies to further improve immunotherapy efficacy and prognosis in solid tumors.

## Data Availability

All data generated or analyzed during this study are included inthis published article.
